# Analysis of Selected Serum Cytokines to Evaluate the Early Efficacy of Benralizumab, Dupilumab, and Mepolizumab in Severe Eosinophilic Asthma Treatment

**DOI:** 10.3390/ijms262010075

**Published:** 2025-10-16

**Authors:** Aleksandra Niemiec-Górska, Łukasz Labus, Sylwia Mielcarska, Joanna Glück, Zenon Czuba, Marcin Cyrnek, Olga Branicka, Barbara Rymarczyk, Radosław Gawlik

**Affiliations:** 1 Chair and Department of Internal Medicine, Allergology and Clinical Immunology, Faculty of Medical Sciences in Zabrze, Medical University of Silesia, Medyków 14 Street, 40-752 Katowice, Polandrgawlik@sum.edu.pl (R.G.); 2 Doctoral School, Medical University of Silesia, 40-752 Katowice, Poland; 3 Department of Lung Diseases and Tuberculosis, Faculty of Medical Sciences in Zabrze, Medical University of Silesia, 40-752 Katowice, Poland; 4 Chair and Department of Medical and Molecular Biology, Faculty of Medical Sciences in Zabrze, Medical University of Silesia, 40-752 Katowice, Poland; 5 Chair and Department of Microbiology and Immunology, Faculty of Medical Sciences in Zabrze, Medical University of Silesia, 40-752 Katowice, Poland; 6 Chair and Department of Internal Medicine and Clinical Pharmacology, Faculty of Medical Sciences in Katowice, Medical University of Silesia, 40-752 Katowice, Poland

**Keywords:** eosinophilic asthma, benralizumab, dupilumab, mepolizumab cytokines

## Abstract

Background: Severe asthma is a chronic, difficult-to-treat disorder that significantly affects quality of life, and oral glucocorticosteroids are usually required. Many patients suffering from severe asthma exhibit T2 inflammation and may benefit from biological treatment. This study aims to evaluate changes in cytokine concentrations during therapy with benralizumab, dupilumab, and mepolizumab in severe eosinophilic asthma. Materials and Methods: In this prospective, single-center study, 39 patients with severe eosinophilic asthma received treatment with one of the above-mentioned biologics. Parameters, such as the cytokine profile (Human Th9/Th17/Th22 Luminex, Performance Assay 18-plex Fixed Panel, R&D Systems, Minneapolis, MN, USA) and additionally the Asthma Control Questionnaire (ACQ), mini-Asthma Quality of Life Questionnaire (mini-AQLQ), spirometry (FEV1, FEV/FVC), FeNO, and functional status, were assessed at baseline and after 3–4 months of therapy. Results: The biologic therapies demonstrated diverse effects on inflammatory biomarkers. Dupilumab showed the most pronounced decreases in CD40L, IL-6, and FeNO in comparison to other drugs. In turn, the greatest decrease in TNF-α concentration was observed in the group treated with mepolizumab. Conclusion: Changes in cytokine concentrations highlight the heterogenous immunomodulatory mechanisms of biologics and support personalized strategies based on inflammatory profiles. However, the results should be interpreted with prudence, as the concentrations of cytokines in blood serum fluctuate and the study sample size is small.

## 1. Introduction

Asthma is a heterogenous, chronic inflammatory disease that impacts the respiratory tract, marked by hyperresponsiveness and variable airway obstruction and symptoms such as wheezing, dyspnea, cough, and chest tightness [1]. Asthma, once regarded as one particular disease, is now acknowledged as a syndrome consisting of several phenotypes and endotypes, which vary in etiology, clinical manifestation, and therapeutic response. This has led to the classification of asthma into two major immunologic endotypes: T2-high and T2-low inflammation [2].

T2-low asthma is characterized by a predominance of Th1- or Th17-driven immune responses, accompanied by the secretion of cytokines such as IL-2, IL-17, IFN-γ, and IL-1β. IL-1β produced by macrophages and dendritic cells promotes inflammation by enhancing leukocyte recruitment and supports Th1 polarization in synergy with IL-12 [3]. Additionally, elevated IFN-γ levels are associated with T2-low asthma and corticosteroid resistance [4].

T2-high asthma is, on the other hand, primarily mediated by type 2 immune responses that involve both innate and adaptive immunity. Principal cellular components comprise T-helper (Th2) cells, group 2 innate lymphoid cells (ILC2), eosinophils, mast cells, basophils, and immunoglobulin E-producing lymphocytes B. These cells initiate a series of inflammatory signals by releasing cytokines, including interleukin-4 (IL-4), IL-5, IL-9, and IL-13, which contribute to eosinophilic inflammation, mucus hypersecretion, and airway remodeling [5].

Biological treatments provide relief by specifically targeting components of the type 2 inflammatory system. Monoclonal antibodies directed at inflammatory pathways are available for the management of severe asthma [6]. Currently approved monoclonal antibodies for severe asthma treatment include anti-IgE (omalizumab), anti-IL5/anti-IL5R (mepolizumab, reslizumab, and benralizumab), anti-IL4R (dupilumab), and anti-TSLP (tezepelumab). Precisely, benralizumab, mepolizumab, and dupilumab are used to treat eosinophilic asthma [7].

In addition to Th1/Th2 cytokines, IL-6, CD40L, TNF-α, IFN-γ, MIP-3α, and IL-10 contribute to asthma-related inflammation. IL-1β and TNF-α induce IL-6, which plays a role in inflammation, autoimmunity, cancer, and asthma [8]. Obesity-related IL-6 activity may worsen lung function and increase asthma exacerbations [9].

TNF-α is a crucial cytokine in the innate immune response. TNF-α dysregulation is connected to inflammatory conditions such asthma, rheumatoid arthritis, and inflammatory bowel diseases [10]. Eosinophil and neutrophil migration, endothelial cell cytotoxicity, T cell activation, and epithelial cell adhesion molecule synthesis are intensified by TNF-α [11,12]. Additionally, TNF-α triggers the release of pro-inflammatory cytokines such as IL-6 and IL-8. Initial studies with small patient populations showed that anti-TNF treatment improved asthma-related quality of life, airway hyperresponsiveness, lung function, and exacerbation frequency [11].

CD40L (CD154) is a co-stimulatory molecule found on activated CD4^+^ T cells and platelets [13]. It interacts with CD40 on B, dendritic, fibroblast, and epithelial/endothelial cells [14]. CD40 and CD40L molecules interact to trigger a specific immune response and enable cellular communication and signaling [15]. This binding promotes the production of adhesion molecules, chemokines, cytokines, tissue factors, reactive oxygen species, various metalloproteinases, growth factors, and other inflammatory mediators [16]. Mast cells expressing CD40L can enhance IgE production by promoting class switching in B cells (with the involvement of IL-4, IL-13, and IL-6), which may exacerbate asthma symptoms [17].

Macrophage inflammatory protein 3-alpha (MIP-3α), also known as CCL20, is crucial to the immune system and inflammatory processes [18]. MIP-3α plays a crucial role in its extensive anti-infective activity [19]. Additionally, the upregulation of MIP-3α is significant in autoimmune conditions like psoriasis, rheumatoid arthritis, and inflammatory bowel disorders [20]. The secretion of MIP-3α is significantly enhanced by epithelial cells following inflammatory stimuli, including IL-1β and TNF-α [21]. In addition, MIP-3α plays a role in the pathogenesis of asthma, especially the pathogenesis associated with T2 inflammation. Interleukins IL-4 and IL-13 influence the increase in MIP-3α secretion by airway epithelial cells [22].

IL-10 is a potent modulatory cytokine that may suppress the production of pro-inflammatory cytokines (IFN-γ and TNFα) by T-helper 1 (Th1) cells. Similarly, IL-10 inhibits T2 inflammation by diminishing the release of pro-inflammatory cytokines from mast cells and reducing allergen-specific IgE production [23].

This study aims to evaluate changes in cytokines concentrations within 3–4 months of the biological treatment of severe eosinophilic asthma with benralizumab, dupilumab, and mepolizumab, Additionally, correlations between cytokine concentrations and clinical parameters were examined, providing insights into the evolving therapeutic influence on inflammatory responses.

## 2. Results

### 2.1. Baseline Patient Characteristics and Their Relevance to Biologic Treatment Efficacy in Severe Bronchial Asthma

**Figure 1:**
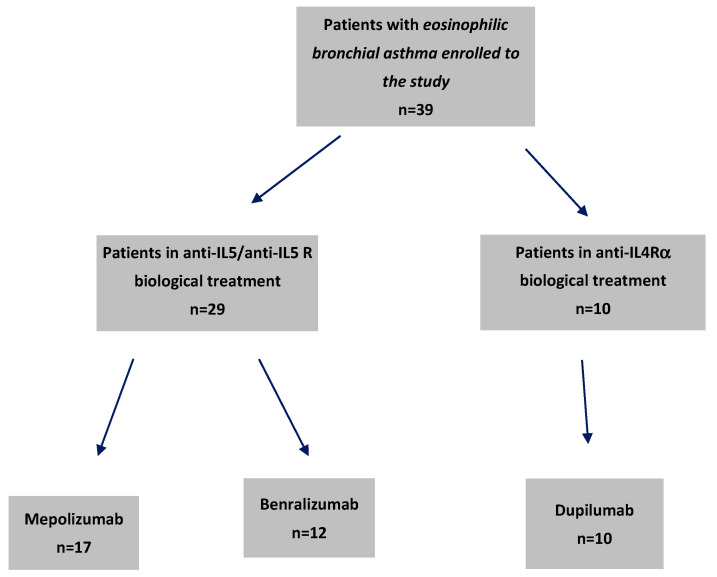
Flow chart of patients receiving biological treatment for severe eosinophilic asthma with particular drugs enrolled on this study.

The cohort under investigation comprises 39 adults, predominantly female (76.9%), diagnosed with severe bronchial asthma. These patients were treated with one of three biologic therapies—benralizumab (*n* = 12), dupilumab (*n* = 10), or mepolizumab (*n* = 17) (Figure 1). The baseline clinical characteristics, as delineated in Table 1, provide a comprehensive profile of the cohort, both overall and stratified by treatment group, offering insights into their demographic and clinical profiles and complete blood counts. In turn, Table 2 shows the initial concentrations of the cytokines studied.

### 2.2. Differences Across Treatment Groups

Statistical comparisons of baseline characteristics reveal no profound differences across the treatment groups, revealing a relatively homogeneous cohort at the outset of biologic therapy. However, allergic status shows a significant difference (*p* = 0.018), indicating that dupilumab patients (90.0% allergic) differ significantly from benralizumab patients (33.3%, *p* < 0.05), but not from mepolizumab patients (47.1%, *p* ≥ 0.05) (Table 1).

### 2.3. Cytokine Profile Analyzed

The analysis of 18 cytokines (CD40L, GM-CSF, IFNγ, IL-1β, IL-2, IL-4, IL-4, IL-5, IL-6, IL-9, IL-10, IL-12p70, IL-13, IL-15, IL-17A, IL-17E, IL-33, MIP-3α, and TNFα) according to the reagent kit was conducted. However, in the post-analysis phase, the results of IL-2, IL-4, IL-5, IL-9, IL-10, IL-12p70, IL-13, IL-17A, IL-17E, and IL-33 were nearly undetectable at both assessment points. Therefore, these cytokines were not considered in the study.

### 2.4. Baseline Correlations of Demographic, Asthma Control, and Pulmonary Function Parameters with Cytokine Profiles in Severe Bronchial Asthma

To further study the data at baseline, in the next step we explored the interplay between baseline clinical characteristics and systemic inflammatory cytokine profiles to elucidate potential mechanistic relationships underlying disease severity and therapeutic response.

**Figure 2:**
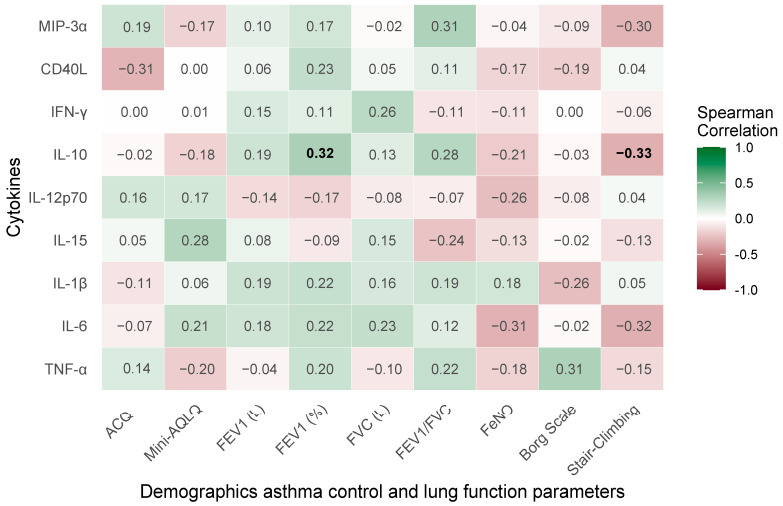
Spearman’s correlation matrix of baseline demographic information, asthma control, lung function parameters, and studied cytokines in patients with severe bronchial asthma. Significant correlations (*p* < 0.05) are marked in bold.

The Spearman correlation matrix (Figure 2) illustrates pairwise associations between clinical parameters and pro-inflammatory or modulatory cytokines (macrophage inflammatory protein-3α [MIP-3α], CD40 ligand [CD40L], interferon-gamma [IFN-γ], interleukin [IL]-10, IL-12p70, IL-15, IL-1β, IL-6, and tumor necrosis factor-alpha [TNF-α]).

The analysis reveals a range of associations both positive and negative, predominantly weak to moderate (Rho ranging from −0.33 to 0.32), reflecting a complex yet subtle interplay between clinical and inflammatory markers. An association is observed between stair-climbing capacity and IL-10 (Rho = −0.33, *p* = 0.049). On the contrary, FEV1% demonstrates a significant positive correlation with IL-10 (Rho = 0.32, *p* = 0.047).

### 2.5. Baseline Concentrations of Serum Cytokines

For most cytokines, no baseline differences were observed between cytokine concentrations in groups of patients eligible for treatment with particular drugs. However, CD40L levels differ significantly (*p* = 0.012), showing dupilumab (median 2573.5 pg/mL) being higher than benralizumab (932.2 pg/mL, *p* < 0.05) and mepolizumab (1146.0 pg/mL, *p* < 0.05). IL-6 levels also differ (*p* = 0.024), with dupilumab (median 2.5 pg/mL) elevated compared with benralizumab (1.0 pg/mL, *p* < 0.05), but not mepolizumab (2.3 pg/mL, *p* ≥ 0.05) (Table 2).

### 2.6. Efficacy Outcomes of Biologic Treatments in Severe Bronchial Asthma: Changes from Baseline to Follow-Up

Table 2 shows the changes in follow-up point after 3–4 months of biologic therapy, reported as medians with 95% confidence intervals (CIs), for cytokine concentrations, alongside within-group *p*-values assessing the significance of changes over time and between-group *p*-values for pairwise comparisons (benralizumab vs. dupilumab, benralizumab vs. mepolizumab, dupilumab vs. mepolizumab). This analysis elucidates the therapeutic impact of each biologic, highlights within-group improvements, and assesses differences in efficacy across treatments.

#### 2.6.1. CD40L

Table 2 reveals significant between-group differences in CD40L changes, with dupilumab exhibiting a pronounced reduction (median −957.0 pg/mL, 95% CI: −1868.0, −187.0, *p* = 0.027) compared with benralizumab. Pairwise Dunn’s test *p*-values (Ben vs. Dup: 0.015, Dup vs. Mep: 0.027) confirm dupilumab’s superior reduction. Table 2 shows that baseline CD40L levels are significantly higher in the dupilumab group (median 2573.5 pg/mL, IQR: 1421.0–3741.5) compared with benralizumab (median 932.2 pg/mL, IQR: 349.8–1650.6) and mepolizumab (median 1146.0 pg/mL, IQR: 641.7–2003.0, *p* = 0.012).

#### 2.6.2. IL-10

A statistically significant decrease in IL-10 concentration was observed during the treatment with dupilumab (median −5.1 ng/mL, 95% CI: −6.6, −1.0, *p* = 0.024) and mepolizumab (median −3.0 ng/mL, 95% CI: −11.2, −0.5, *p* = 0.015). No significant between-group differences were observed.

#### 2.6.3. IL-6

IL-6 shows a significant between-group difference (Ben vs. Dup: *p* = 0.045), with dupilumab achieving a near-significant reduction (median −0.7 pg/mL, 95% CI: −1.5, 0.0, *p* = 0.059). No statistically significant differences were observed for benralizumab and mepolizumab. Table 2 reveals significantly higher baseline IL-6 in the dupilumab group (median 2.5 pg/mL, IQR: 1.6–3.0) compared with benralizumab (median 1.0 pg/mL, IQR: 0.5–1.4, *p* = 0.024), with mepolizumab showing intermediate results (median 2.3 pg/mL, IQR: 0.8–3.4).

#### 2.6.4. TNF-α

TNF-α changes demonstrate significant between-group differences (Ben vs. Mep: *p* = 0.008, Dup vs. Mep: *p* = 0.045). Table 2 shows that baseline TNF-α levels are slightly higher in the mepolizumab group (median 3.4 pg/mL, IQR: 2.2–4.3) compared with benralizumab (median 2.3 pg/mL, IQR: 1.0–3.1) and dupilumab (median 2.4 pg/mL, IQR: 0.8–4.3), with no significant baseline difference (*p* = 0.224).

#### 2.6.5. IL-12p70

We observed an increase in IL-12p70 in all groups; however, statistical significance applies to patients as a whole (median 0.5 pg/mL, 95% CI: 0.0, −0.80, *p* = 0.032), but not to individual groups of patients treated with specific drugs. No significant between-group differences were observed.

### 2.7. Clinical Parameter Changes

Similarly, Table 3 presents changes at the follow-up point after 3–4 months of biological therapy, but for clinical parameters such as ACQ, mini-AQLQ, FEV1%, FeNO, and the subjective assessment of exercise tolerance.

A statistically significant improvement during biological treatment was observed for the following clinical parameters: ACQ, mini-AQLQ, perceived breathlessness assessed using the modified Borg scale, and stair-climbing capacity. All groups achieved significant within-group improvements (*p* < 0.05) but no between-group differences.

### 2.8. Correlations of Treatment-Induced Changes in Clinical and Pulmonary Function Parameters with Cytokine Profiles in Severe Bronchial Asthma

**Figure 3:**
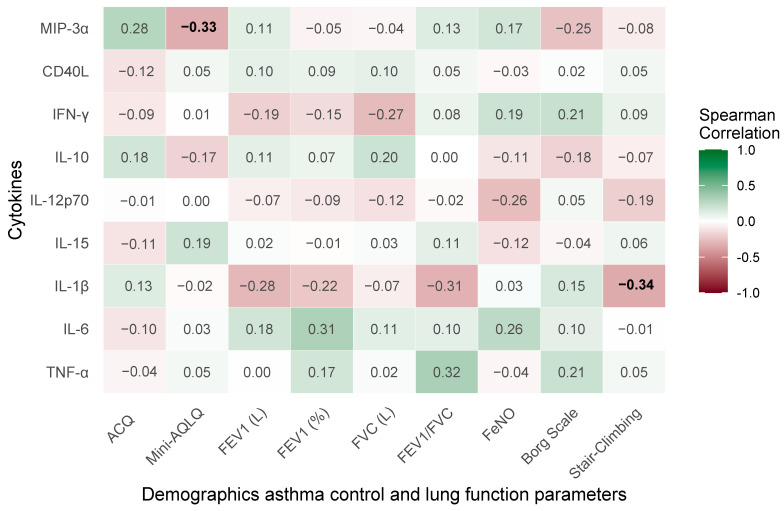
Spearman’s correlation matrix of treatment-induced changes in clinical and pulmonary function parameters with cytokine profiles in severe bronchial asthma. Significant correlations (*p* < 0.05) are marked in bold.

The current section examines the relationships between treatment-induced changes in clinical and pulmonary function parameters and cytokine profiles following 3–4 months of therapy with benralizumab, dupilumab, or mepolizumab, with reporting in the form of Spearman’s correlation matrix in Figure 3.

Two correlations achieve statistical significance (*p* < 0.05), underscoring clinically relevant relationships. Stair-climbing capacity exhibits a significant negative correlation with IL-1β (Rho = −0.34, *p* = 0.045). The mini-AQLQ demonstrates a significant negative correlation with MIP-3α (Rho = −0.33, *p* = 0.042), indicating that enhanced quality of life aligns with reduced MIP-3α levels.

## 3. Discussion

This prospective study evaluated clinical, functional, and laboratory variables in a cohort of severe asthma patients prior to and during therapy with benralizumab, dupilumab, and mepolizumab. The baseline characteristics of patients receiving biologic treatment showed no substantial differences between the groups.

This study presents novel insights into the unique immunomodulatory characteristics of benralizumab, dupilumab, and mepolizumab in individuals with severe bronchial asthma, emphasizing their various impacts on inflammatory biomarkers. Our findings highlight the variability of immune responses in asthma and indicate that some biologics may selectively target distinct inflammatory pathways.

The most significant decreases in CD40L and IL-6 were noted in the dupilumab cohort, which may be attributable to elevated baseline levels of these markers. According to Tian et al., CD40L engagement on epithelial cells enhances asthma severity, but decreasing CD40 gene expression in these cells led to less disease progression [24]. Although not concerning the treatment of asthma but allergic bronchopulmonary mycosis (ABPM) instead, Tashiro et al. described the beneficial effect of dupilumab on reducing CD40L levels [25].

Dupilumab has demonstrated efficacy in decreasing IL-6 concentrations in nasal fluid among individuals with aspirin-exacerbated respiratory disease (AERD) [26]. Not only does dupilumab have an effect on diminishing levels of IL-6, but mepolizumab also induces this effect. According to Malik et al., treatment with mepolizumab reduced the proliferative capacity of ILC2 and was associated with a lower release of pro-inflammatory cytokines, such as IL-6 [27]. In our study, we observed a decrease in IL-6 levels only for dupilumab. No significant changes were found for the other two drugs.

In our research, mepolizumab resulted in the most significant reduction in TNF-α. Surprisingly, neither dupilumab nor benralizumab lowered TNF-α concentrations. Mepolizumab demonstrated statistically significant superiority in this regard compared with the other two drugs. In turn, in a study performed by Rogaliani et al., both mepolizumab and benralizumab decreased TNF-α levels [28].

Interleukin (IL)-10 is a significant immunomodulatory cytokine, since it downregulates the production of pro-inflammatory Th2 cytokines and supports the resolution of inflammation [29]. Indeed, both steroid treatment and allergen-specific immunotherapy are recognized to increase endogenous IL-10 levels [30]. In turn, according to Howell et al., mepolizumab had a weaker effect on IL-10 level than OCS [31]. Furthermore, studies have demonstrated inconsistent findings concerning IL-10 levels during dupilumab therapy. Čelakovska et al. observed a markedly elevated plasma concentration of IL-10 in patients with atopic dermatitis treated with dupilumab compared with the control group [32]. However, Harb et al. explained that, in the absence of IL-4R signaling, diminished IL-10 synthesis results from the insufficient growth of an IL-10+ TH2 population, rather than a global deficiency in IL-10 production by CD4+ T cells [33]. Interestingly, in the case of benralizumab, Bergantini et al. observed a decrease in IL-10 concentration after 1 month of treatment, while after 6 months of therapy, an increase in IL-10 levels was found [34]^.^ Surprisingly, our investigation revealed that the concentration of IL-10 diminished after 3–4 months of therapy with all assessed biological agents.

IL-12p70 is the active heterodimeric form of interleukin 12. IL-12 is a pro-inflammatory cytokine and it is crucial for both innate resistance and acquired immunity, since it stimulates the generation of IFN-γ from NK and NKT cells during the initial stages of the immune response and promotes the development of Th1 cells [35]. IL-12 stimulates the Th1 immune response and decreases the Th2 response. In eosinophilic asthma, Th2 responses dominate, resulting in diminished levels of IL-12 [36]. In an animal study providing IL-12 to mice previously exposed to an antigen, airway hyperresponsiveness and pulmonary eosinophilia were eliminated, and the expression of IL-4 and IL-5 was diminished [36]. The impact of biological treatment on eosinophilic asthma concerning the IL12p70 profile remains inadequately researched. In our experiment, we observed an increase in IL-12p70 in all groups treated with dupilumab, benralizumab, and mepolizumab. However, statistical significance applied to patients as a whole, but not to individual groups of patients treated with particular drugs.

Additionally, our study showed a negative correlation between MIP-3α and the mini-AQLQ, which means that a lower concentration of MIP-3α correlates with a better quality of life. MIP-3α concentrations are modulated by pro-inflammatory cytokines, including IL-1β and TNF-α, as well as pro-allergic cytokines such as IL-4 and IL-13 [22]. What is essential is that MIP-3α/CCL20 primarily attracts Th17 cells and is associated with neutrophilic inflammation and severe asthma phenotypes, suggesting that the efficacy of these three biologics may be partially influenced by the inhibition of Th17-associated inflammatory pathways. Park et al. [37,38] found that MIP-3α induced airway hyperresponsiveness and bronchial airway remodeling, whereas MIP-3α inhibitors and monoclonal antibodies could reduce airway hyperresponsiveness, airway inflammation, and airway remodeling caused by MIP-3α [39]. In turn, bronchial hyperresponsiveness and more severe inflammation are associated with more pronounced symptoms and a poorer quality of life [40].

We observed also a negative correlation between IL-1β concentration and stair-climbing capacity. This interleukin is highly pro-inflammatory and is reported to influence the severity of inflammation in the airways. According to Kim et al., IL-1β is associated with decreased asthma control and FEV1 [41]. In turn, impaired lung function is related to a reduced exercise capacity.

However, no significant correlations were observed between cytokine concentrations and lung function parameters. Cytokines, especially IL-10 and IL-12, are now recognized to have short half-lives and exhibit variable stability in circulation [42]. Therefore, cytokine concentrations may vary between measurement points and may not necessarily be related to changes in clinical status. Furthermore, cytokine concentrations in the blood do not always reflect their concentrations in tissues [43]. In instances of respiratory tract inflammation, measuring cytokines in bronchoalveolar lavage fluid would be significantly more reliable [44].; this path of scientific research should be considered in the future. However, it is important to note that the procedure would be more invasive.

Our study confirms the high efficacy of biological drugs in asthma therapy. Treatment with all analyzed drugs, i.e., benralizumab, dupilumab, and mepolizumab, results in significant clinical improvement, expressed as an increase in mini-AQLQ scores and a decrease in ACQ scores. Improvements in questionnaire scores (a decrease in ACQ and an increase in mini-AQLQ) during biological treatment is a well-known phenomenon described in many scientific studies, but it is also an indicator of the effectiveness of therapy and a condition for continuing treatment [45,46,47]. Furthermore, we observed a notable improvement in exercise tolerance, both in terms of perceived breathlessness (Borg’s scale) and exercise capacity (stair-climbing). To date, there are few publications on the improvement of physical exercise capacity during the biological treatment of severe asthma [48,49,50].

Fractional exhaled nitric oxide (FeNO) is an economical, easy to measure biomarker used for the identification of type 2 airway inflammation. Elevated FeNO levels are observed in individuals with acute or chronic airway inflammation, including asthma. FeNO may also be utilized to forecast the response to inhaled corticosteroids, assess adherence, and determine the efficacy of biologic treatment [51]. In our study, dupilumab diminished FeNO levels, aligning with its suppression of IL/4/IL-13, a principal regulator of nitric oxide synthase production in the airway epithelium [51]. However, the decrease is not statistically significant. Mepolizumab resulted in a slight reduction in FeNO, whereas benralizumab, despite its anti-eosinophilic properties, was linked to an elevation in FeNO levels. The last result, probably, is random and has no scientific explanation. According to scientific studies, benralizumab slightly reduces or has no effect on FeNO results [52].

### Limitations of the Study

The main limitation of this study is the short observational period and only one follow-up point. However, due to logistical and financial constraints it was designed to include only two assessment points.

In addition, the exacerbation rate during biological treatment was not analyzed due to the short observation period of 3–4 months.

A further problem is the limited size of the research cohort, which may only partially represent the outcomes found in the wider group of patients undergoing biological therapy. Also, a small sample size results in a diminished statistical power, challenging the identification of significant differences unless they are substantial. The issue is further complicated by potential imbalances in baseline characteristics between groups. Initially higher parameter values in certain groups may create the illusion of enhanced improvement; therefore, conclusions should be formulated with prudence.

Another issue is the lack of a placebo control group. Our research is based on a real-life study. As observed in other studies of this type using biological therapy, different situations also occur. Nonetheless, we are aware that the absence of a control group diminishes the reliability of the comparative data obtained.

Moreover, for logistical reasons, the 6 min walk test, a leading functional test for assessing exercise capacity and endurance in patients with lung diseases, was not performed. We concentrated solely on subjective feelings, such as dyspnea, and patient reports on factors that affect patients’ quality of life.

## 4. Materials and Methods

### 4.1. Study Design and Participants

This single-center, prospective, real-life study was performed from April 2023 to September 2024 at the Department of Internal Diseases, Allergology, and Clinical Immunology in the University Clinical Hospital named the K. Gibiński Medical University of Silesia in Katowice, Poland. This study included 39 patients diagnosed with severe eosinophilic asthma who met the eligibility criteria for treatment at the time of enrolment; 17 of them started treatment with mepolizumab, 12 with benralizumab, and 10 with dupilumab. Reslizumab was omitted due to its unavailability in Poland.

### 4.2. Inclusion Criteria

This study included patients who met the criteria for initiating the biologic treatment of severe asthma with benralizumab, dupilumab, or mepolizumab in accordance with the requirements that are mandatory in Poland.

The eligibility criteria for the biological treatment of eosinophilic asthma according to the Polish Ministry of Health guidelines are as follows:

- Blood eosinophil count ≥ 350/µL in the last 12 months or ≥150 cells/μL if systemic glucocorticosteroids at a dose ≥ 5 mg per day had to be taken systematically in the 6 months prior to the qualification and the cumulative annual dose of oral glucocorticosteroids was ≥1.0 g (calculated as prednisone) due to a lack of asthma control.
- The need for high doses of inhaled glucocorticosteroids (>1000 mcg of beclomethasone dipropionate per day or another inhaled glucocorticosteroid at an equivalent dose determined according to current guidelines from The Global Initiative for Asthma (GINA)) in combination with another asthma control medication.
- Two or more exacerbations in the past year that required systemic glucocorticosteroids or an increase in their dose for more than three days in people who use them chronically.
- The patients met at least two of the following criteria:
  (a) Symptoms of uncontrolled asthma (lack of asthma control in the ACQ (Asthma Control Questionnaire) > 1.5 points).
  (b) Hospitalization in the last 12 months due to asthma exacerbation.
  (c) A life-threatening asthma attack incident in the past.
  (d) Persistent airway obstruction (first-second expiratory volume FEV1 < 80% of normal value or diurnal variation in peak expiratory flow PEF > 30%).
  (e) Impaired quality of life due to asthma (mean score on the asthma quality of life test mini-AQLQ < 5.0 points).
- The exclusion of other hypereosinophilic syndromes.
- Non-smoking.
- The exclusion of other clinically relevant pulmonary diseases [26].

According to the summary of product characteristics, dupilumab was administered every two weeks at a dose of 200 mg or 300 mg if the patient had moderate to severe atopic dermatitis or severe chronic rhinosinusitis with nasal polyps; mepolizumab was administered at a dose of 100 mg every four weeks; while benralizumab was initially administered three times every four weeks at a dose of 30 mg, and subsequently every eight weeks.

### 4.3. Exclusion Criteria

The criterion for exclusion from this study was the discontinuation of treatment before the follow-up point after 3–4 months of treatment due to side effects or patient wishes.

### 4.4. Assessment of Clinical Efficacy

The seven-item Asthma Control Questionnaire (ACQ-7) assessed asthma control. Five questions concern symptoms, one concerns rescue bronchodilators, and one concerns FEV1. Each task is graded on a seven-point scale from 0 (full control) to 6 (highest impairment). Averaging the questions yields the ACQ score. ACQ changes of 0.5 are regarded minimally noteworthy.

The mini-AQLQ was used to assess asthma-related quality of life. The mini-AQLQ comprises 15 items on a 7-point scale, with 1 indicating considerable impairment and 7 indicating no impairment. A higher questionnaire score increases quality of life. The minimum relevant mini-AQLQ change is 0.5.

### 4.5. Laboratory Tests

All the patients enrolled in this study underwent routine complete blood tests at the time of admission for biological therapy (point 0). Samples were collected using a test tube containing an anticoagulant (EDTA—ethylenediaminetetraacetic acid). The Sysmex XN-350 hematology analyzer, manufactured by Sysmex Europe Corporation (Norderstedt, Germany), measured and recorded the whole blood count. The eosinophil count in the study may have been lower than 350/µL because the morphological result on the day of admission was taken into account. A result of more than 350 eosinophils in the last 12 months (historical determination) is acceptable to initiate biological treatment as well. We assumed that all determinations in the study were performed on the same laboratory equipment.

Additionally, CRP was measured at point 0. Normality was set at 5 mg/L.

### 4.6. Evaluation of the Cytokine Screening Panel

An additional blood sample for cytokine panel analysis was obtained after admission for biological therapy (point 0). Whole blood samples were allowed to coagulate at ambient temperature for 30 min. The supernatant underwent centrifugation at 5000× *g* for 10 min. Subsequently, serum samples were preserved at −80 °C until analysis. A follow-up blood sample was taken after 3–4 months of biological therapy (point 1) to assess the variation in the concentration of selected cytokines (CD 40 Ligand, GM-CSF, IFN-γ, IL-1β, IL-2, IL-4, IL-5, IL-6, IL-9, IL-10, IL-12p70, IL-13, IL-15, IL-17A, IL-17E, IL-33, MIP-3α, and TNFα).

Prior to the examination, the samples were centrifuged for 5 min. A multiplex test (Human Th9/Th17/Th22 Luminex, Performance Assay18-plex Fixed Panel, R&D Systems, Minneapolis, MN, USA) was used to determine cytokine concentrations. A 50 µL aliquot of blood serum was diluted 1:2 with a sample diluent, treated with antibody-conjugated beads, biotinylated secondary antibodies, and subsequently with streptavidin–phycoerythrin. Standard curves for each analyzed molecule were generated using the appropriate cytokine standard solutions. The beads were measured using the Luminex System. The intra-assay %CV fluctuated up to 15%, whereas the inter-assay %CV varied to 25%, contingent upon the protein analyzed.

### 4.7. Allergy Detection

Allergy was diagnosed on the basis of allergen-specific IgE antibody determinations in the serum of patients using the Polycheck method. A concentration > 0.35 kU/L was considered a positive result A second allergy diagnosis method was skin prick tests. A positive test for IgE-mediated hypersensitivity is a 3 mm urticarial wheal with erythema.

### 4.8. Spirometry

Spirometry was routinely performed in patients before the administration of the biologic treatment and prior to the of use of a bronchodilator (point 0) and after 3–4 months of treatment (point 1). The test was performed by the nurses in the Allergology Department using a Spiro Scout spirometer (Ganshorn Medizin Electronic, Niederlauer, Germany). The following parameters were evaluated: FEV1 (forced expiratory volume), FEV1%, FVC (forced vital capacity), and FEV1/FVC.

### 4.9. Fractional Exhaled Nitric Oxide (FeNO)

Furthermore, a FeNO test measuring the concentration of nitric oxide in breathed air was performed on patients during the trial. The assessment was carried out using a Medisoft device (Ganshorn, Germany) prior to the start of biological therapy and during the follow-up visit after 3–4 months of treatment.

### 4.10. Assessment of Functional Status and Exercise Tolerance

A subjective assessment of exertional dyspnea according to the modified Borg scale (0–10 points), assessed subjectively according to the possibility of climbing a particular floor by stairs, was performed.

### 4.11. Evaluation

The evaluation conducted after 3–4 months of therapy was related to the necessity of the initial assessment of treatment efficacy. In compliance with the requirements of the drug program in Poland, spirometry and evaluations of ACQ and mini-AQLQ should be performed at this point. The evaluation was subsequently broadened to include exercise tolerance, FeNO, and cytokine profile assessment. The evaluation after 3 months was performed for dupilumab and mepolizumab; however, for benralizumab, it was carried out after 4 months due to the medication administration schedule.

### 4.12. Ethics

The study protocol was reviewed and approved by the Bioethics Committee at the Medical University of Silesia in Katowice (BNW/NWN/0052/KB1/18/I/23) and was in accordance with the ethical principles for human experimentation initiated by the Declaration of Helsinki.

### 4.13. Statistical Analysis

Statistical significance was determined by a two-tailed *p*-value < 0.05, with near-significant trends noted at *p* < 0.10, reflecting the exploratory character of the study. Based on Shapiro–Wilk’s tests, all studies assumed a non-normal data distribution, requiring non-parametric approaches.

For continuous variables, medians with interquartile ranges (IQRs) were reported for baseline characteristics (Table 2 and Table 3) and medians with 95% CIs for changes over time (Table 2 and Table 3), reflecting the central tendency and variability in this small cohort. Categorical variables were summarized as frequencies and percentages with non-missing counts for missing data. To account for skewness and small sample sizes, non-parametric bootstrap calculations with 1000 resampling iterations generated confidence ranges for continuous changes.

Kruskal–Wallis’s rank sum tests for continuous variables and Fisher’s exact tests for categorical variables were used to compare baselines among treatment groups (benralizumab, dupilumab, and mepolizumab). For significant continuous variables (*p* < 0.05), Dunn’s test with Holm’s correction was used for post hoc pairwise comparisons to control family-wise error rates. Pairwise Fisher’s exact tests with FDR correction were used for categorical variables. Post hoc analyses were conducted using a letter-based system, with groups with similar letters indicating no significant difference (*p* ≥ 0.05 after correction) and groups with dissimilar letters indicating significant differences (*p* < 0.05).

For paired data, clinical parameter changes from baseline to 3–4 month follow-up were computed as post treatment minus baseline. Wilcoxon’s signed-rank test assessed the within-group significance of these changes. The Kruskal–Wallis test and Dunn’s test were used to compare changes across groups (benralizumab vs. dupilumab, dupilumab vs. mepolizumab).

Spearman’s correlation analysis assessed non-parametric relationships between variables. The t statistic was approximated asymptotically to estimate *p*-values. No multiple comparison corrections were used due to the small sample size, extensive correlation analysis parameters, and the exploratory objective of creating hypotheses rather than conclusive effects.

Statistical significance was defined as a two-tailed *p*-value less than 0.05, with near-significant trends noted at *p* < 0.10, acknowledging the exploratory nature of this investigation. All analyses assumed a non-normal distribution of data, as assessed through Shapiro–Wilk’s tests, prompting the use of non-parametric methods.

Descriptive statistics for continuous variables were reported as medians with interquartile ranges (IQRs) for baseline characteristics and medians with 95% confidence intervals (CIs) for changes over time reflecting the central tendency and variability within this small cohort. Categorical variables were summarized as frequencies and percentages, with non-missing counts specified for variables exhibiting missing data. Confidence intervals for continuous changes were estimated using a non-parametric bootstrap approach with 1000 resampling iterations, to account for skewness and small sample sizes.

Baseline comparisons across treatment groups (benralizumab, dupilumab, mepolizumab) were conducted using the Kruskal–Wallis rank sum test for continuous variables and Fisher’s exact test for categorical variables. For continuous variables with significant overall *p*-values (*p* < 0.05), post hoc pairwise comparisons were performed using Dunn’s test with Holm’s correction to control the family-wise error rate within each parameter. For categorical variables, pairwise Fisher’s exact tests with false discovery rate (FDR) correction were applied. The results of post hoc analyses were reported using a letter-based system, where groups sharing the same or overlapping letters indicate no significant difference (*p* ≥ 0.05 after correction), while groups with distinct letters denote significant differences (*p* < 0.05 after correction).

Changes in clinical parameters from baseline to 3–4 month follow-up were calculated as the difference (post treatment minus baseline) for paired observations. The within-group significance of these changes was evaluated using the Wilcoxon signed-rank test. Between-group differences in changes were analyzed using the Kruskal–Wallis test, followed by Dunn’s test for pairwise comparisons (benralizumab vs. dupilumab, benralizumab vs. mepolizumab, dupilumab vs. mepolizumab).

Correlation analyses were performed by employing the Spearman method to assess non-parametric associations between variables. *p*-values were estimated using an asymptotic approximation of the t statistic. Given the constrained sample size, the extensive array of parameters in the correlation analysis, and the exploratory objective of generating hypotheses rather than establishing conclusive effects, no adjustments for multiple comparisons were implemented.

#### Characteristics of the Statistical Tool and List of Applied External Libraries

Analyses were conducted using the R Statistical language (version 4.3.3; R Core Team, 2024) on Windows 11 Pro 64 bit (build 26,100), using the packages *boot* (version 1.3.29).

## 5. Conclusions

Benralizumab, dupilumab, and mepolizumab are highly effective biological agents for the management of severe asthma. They promote a reduction in pro-inflammatory cytokines, especially observed during dupilumab therapy. Nevertheless, due to the small sample size and unstable cytokine concentrations in blood serum, the results should be interpreted with caution.

Furthermore, a considerable clinical improvement was found characterized by a drop in ACQ scores, an increase in mini-AQLQ scores, and enhanced exercise tolerance. Furthermore, dupilumab exhibited the most notable reduction in FeNO among the evaluated drugs; however, it was not a statistically significant result.

## Figures and Tables

**Table 1:** Baseline clinical characteristics of patients with severe bronchial asthma by biologic treatment group.

Characteristic	Overall (*n* = 39)	Benralizumab (*n* = 12)	Dupilumab (*n* = 10)	Mepolizumab (*n* = 17)	*p*
Demographics					
Age (years)	56.0 (51.0, 64.0)	57.0 (51.0, 64.5)	55.0 (50.0, 63.0)	54.0 (51.0, 64.0)	0.931
Sex					0.159
Female	30 (76.9%)	7 (58.3%)	8 (80.0%)	15 (88.2%)	
Male	9 (23.1%)	5 (41.7%)	2 (20.0%)	2 (11.8%)	
Clinical Characteristics					
Oral Corticosteroid Use (days/year)	21.0 (12.0, 80.0)	14.0 (11.0, 55.0)	23.0 (15.0, 40.0)	25.0 (19.0, 120.0)	0.725
Disease Duration					0.760
Over 10 years	25 (64.1%)	7 (58.3%)	6 (60.0%)	12 (70.6%)	
Up to 10 years	14 (35.9%)	5 (41.7%)	4 (40.0%)	5 (29.4%)	
Comorbidities					
Hypertension	21 (63.6%) *n* = 33	8 (72.7%) *n* = 11	6 (60.0%)	7 (58.3%) *n* = 12	0.815
Diabetes Mellitus	7 (21.2%) *n* = 33	4 (36.4%) *n* = 11	2 (20.0%)	1 (8.3%) *n* = 12	0.275
Dyslipidemia	12 (36.4%) *n* = 33	4 (36.4%) *n* = 11	4 (40.0%)	4 (33.3%) *n* = 12	1.000
Osteoporosis	5 (13.5%) *n* = 37	2 (16.7%)	2 (20.0%)	1 (6.7%) *n* = 15	0.586
Coronary Artery Disease	3 (9.1%) *n* = 33	1 (9.1%) *n* = 11	0 (0.0%)	2 (16.7%) *n* = 12	0.758
Obstructive Sleep Apnea	12 (33.3%) *n* = 36	5 (41.7%)	4 (44.4%) *n* = 9	3 (20.0%) *n* = 15	0.439
Allergy	21 (53.8%)	4 (33.3%) ^A^	9 (90.0%) ^B^	8 (47.1%) ^AB^	**0.018**
Nasal Polyps	18 (46.2%)	4 (33.3%)	3 (30.0%)	11 (64.7%)	0.128
Complete Blood Count, (×10^3^ cells/µL)					
White Blood Cell Count	8.3 (6.6, 10.1) *n* = 37	9.2 (6.7, 11.4)	7.4 (5.6, 9.5)	8.4 (6.7, 10.1) *n* = 15	0.439
Eosinophil Count	0.4 (0.2, 0.7) *n* = 38	0.4 (0.3, 0.8)	0.2 (0.2, 0.4)	0.5 (0.2, 0.8) *n* = 16	0.219
Neutrophil Count	4.7 (3.7, 6.0) *n* = 37	5.5 (3.9, 7.7)	4.2 (2.9, 5.4)	4.7 (3.7, 5.8) *n* = 15	0.478
Lymphocyte Count	2.0 (1.7, 2.4) *n* = 37	1.9 (1.5, 2.4)	2.0 (1.9, 2.4)	2.2 (1.7, 2.4) *n* = 15	0.596
Additional blood parameters					
Total IgE (IU/mL)	182.0 (34.9, 558.0) *n* = 35	156.0 (125.0, 226.0) *n* = 9	558.0 (182.0, 714.0) *n* = 9	125.0 (32.0, 250.0)	0.194
C-Reactive Protein (mg/L)	2.2 (1.1, 6.2) *n* = 38	1.2 (0.7, 5.5)	3.4 (2.0, 8.7)	2.1 (0.9, 4.5) *n* = 16	0.124

**Table 2:** Baseline values and changes within cytokine concentrations at 3–4 month follow-up compared to baseline in patients with severe bronchial asthma by biologic treatment.

Baseline						Changes After Treatment						
Cytokines (pg/mL)	Overall (*n* = 39)	Benralizumab (*n* = 12)	Dupilumab (*n* = 10)	Mepolizuamb (*n* = 17)	*p*-Value	Overall (*n* = 39)	Benralizumab (*n* = 12)	Dupilumab (*n* = 10)	Mepolizumab (*n* = 17)	*p*-Value		
										Ben vs. Dup	Ben vs. Mep	Dup vs. Mep
MIP-3α	3.3 (2.0, 5.8)	2.5 (1.2, 7.0)	4.4 (3.1, 6.3)	3.3 (2.0, 5.7)	0.333	−0.7 (−1.6, 0.3) *p* = 0.192	0.6 (−2.1, 3.7) *p* = 0.677	−1.6 (−3.7, 0.2) *p* = 0.105	−0.7 (−2.4, 0.9) *p* = 0.224	0.089	0.234	0.178
CD40L	1359.9 (810.0, 2526.6)	932.2 (349.8, 1650.6) ^A^	2573.5 (1421.0, 3741.5) ^B^	1146.0 (641.7, 2003.0) ^A^	**0.012**	−149.0 (−560.0, 380.0) *p* = 0.494	−155.0 (−430.0, 1240.0) *p* = 0.424	−957.0 (−1868.0, −187.0) *p* = **0.027**	277.0 (−639.0, 1246.0) *p* = 0.306	**0.015**	0.089	**0.027**
IFN-γ	0.5 (0.3, 1.2)	0.5 (0.2, 1.2)	0.5 (0.3, 1.0)	0.4 (0.4, 1.4)	0.603	−0.1 (−0.3, 0.0) *p* = 0.105	−0.1 (−0.3, 0.3) *p* = 0.756	−0.0 (−3.5, 0.4) *p* = 0.721	−0.2 (−1.8, 0.0) *p* = 0.064	0.456	0.567	0.612
IL-10	8.5 (6.5, 12.5)	7.5 (2.5, 10.5)	10.5 (8.5, 12.5)	8.5 (6.5, 12.5)	0.215	−3.0 (−5.1, −1.1) *p* = **0.002**	−2.0 (−6.1, 3.9) *p* = 0.564	−5.1 (−6.6, −1.0) *p* = **0.024**	−3.0 (−11.2, −0.5) *p* = **0.015**	0.178	0.345	0.267
IL-12p70	0.3 (0.2, 0.7)	0.3 (0.2, 1.0)	0.3 (0.3, 1.3)	0.3 (0.2, 0.7)	0.725	0.5 (0.0, 0.8) *p* = **0.032**	0.3 (−1.0, 1.1) *p* = 0.529	0.6 (0.3, 1.2) *p* = 0.181	0.5 (−0.5, 1.0) *p* = 0.124	0.623	0.701	0.789
IL-15	1.0 (0.7, 1.3)	1.0 (0.7, 1.3)	0.9 (0.5, 1.2)	1.0 (0.7, 1.3)	0.684	−0.1 (−0.2, 0.1) *p* = 0.252	−0.0 (−0.4, 0.3) *p* = 1.000	−0.2 (−0.2, 0.0) *p* = 0.065	−0.1 (−0.3, 0.2) *p* = 0.569	0.512	0.623	0.734
IL-1β	0.0 (0.0, 0.3)	0.2 (0.0, 0.4)	0.3 (0.0, 0.5)	0.0 (0.0, 0.2)	0.317	0.0 (−0.1, 0.1) *p* = 0.891	0.0 (−0.2, 0.2) *p* = 1.000	−0.1 (−0.3, 0.2) *p* = 0.343	0.0 (−0.1, 0.2) *p* = 0.437	0.456	0.567	0.612
IL-6	1.6 (0.8, 3.0)	1.0 (0.5, 1.4) ^A^	2.5 (1.6, 3.0) ^B^	2.3 (0.8, 3.4) ^AB^	**0.024**	0.0 (−0.5, 0.4) *p* = 0.807	0.2 (−0.4, 0.9) *p* = 0.247	−0.7 (−1.5, 0.0) *p* = 0.059	0.0 (−1.5, 0.9) *p* = 0.798	**0.045**	0.234	0.089
TNF-α	3.1 (1.1, 4.3)	2.3 (1.0, 3.1)	2.4 (0.8, 4.3)	3.4 (2.2, 4.3)	0.224	0.0 (−0.6, 0.5) *p* = 0.916	0.8 (−0.2, 2.2) *p* = 0.110	0.2 (−1.6, 1.2) *p* = 0.695	−0.9 (−1.9, 0.0) *p* = 0.050	0.178	**0.008**	**0.045**

Notes: Continuous variables are presented as median values (Q1, Q3). Changes are calculated as the difference between follow-up and baseline and presented as median values (95% CI) for continuous variables; *n* indicates non-missing paired observations (if different from group sample); *p*-values report the significance of changes within sample (group) over time. *p*-values for pairwise comparisons (benralizumab vs. dupilumab, benralizumab vs. mepolizumab, dupilumab vs. mepolizumab) are unadjusted and calculated using Dunn’s test with Holm’s correction applied. Results of post hoc analyses are reported using a letter-based system, where treatment groups sharing the same (or overlapped, e.g., A vs. AB, B vs. AB) uppercase letter do not differ significantly (*p* ≥ 0.05 after correction), and groups without overlapped (e.g., A vs. B) uppercase letters indicate significant differences (*p* < 0.05 after correction). Abbreviations: MIP-3α = macrophage inflammatory protein-3α; CD40L = CD40 ligand; IFN-γ = interferon-gamma; IL = interleukin; TNF-α = tumor necrosis factor-alpha. Bold numbers indicate statistically significant values.

**Table 3:** Baseline values and changes in clinical indicators at the 3–4 month follow-up relative to baseline in patients with severe bronchial asthma undergoing biologic therapy.

Baseline						Changes After Treatment						
Characteristic	Overall (*n* = 39)	Benralizumab (*n* = 12)	Dupilumab (*n* = 10)	Mepolizumab (*n* = 17)	*p* Value	Overall (*n* = 39)	Benralizumab (*n* = 12)	Dupilumab (*n* = 10)	Mepolizumab (*n* = 17)	*p*-Value		
										Ben vs. Dup	Ben vs. Mep	Dup vs. Mep
**Asthma Control and Quality of Life**												
Asthma Control Questionnaire Score	3.4 (2.9, 4.0)	3.4 (3.2, 4.1)	3.4 (2.6, 3.6)	3.4 (2.7, 4.0)	0.686	−1.1 (−1.4, −0.8) *n* = 38, *p* < **0.001**	−1.0 (−1.7, −0.6) *n* = 11, *p* = **0.004**	−1.2 (−1.9, −0.8) *p* = **0.002**	−1.0 (−1.6, −0.4) *p* = **0.003**	0.612	0.789	0.705
Mini-Asthma Quality of Life Questionnaire Score	2.9 (2.4, 3.4)	2.7 (2.4, 3.4)	3.1 (2.7, 3.4)	2.7 (2.1, 3.5)	0.452	1.1 (0.7, 1.5) *n* = 38, *p* < **0.001**	1.3 (0.7, 2.1) *n* = 11, *p* = **0.008**	1.1 (0.6, 1.8) *p* = **0.002**	0.9 (0.1, 1.7) *p* = **0.015**	0.523	0.456	0.612
**Lung Function**												
FEV1 (% Predicted)	64.0 (52.0, 75.0)	67.0 (50.0, 74.5)	55.5 (51.0, 66.0)	69.0 (53.0, 79.0)	0.515	10.5 (4.5, 16.5) *n* = 37, *p* = **0.002**	8.0 (−7.0, 16.5) *p* = 0.208	10.5 (−0.5, 19.5) *p* = 0.059	15.5 (−2.5, 28.0) *n* = 15, *p* = 0.079	0.567	0.432	0.523
FeNO (ppb)	23.0 (7.0, 63.0) *n* = 27	13.5 (10.0, 63.0) *n* = 10	25.0 (7.0, 35.0) *n* = 7	34.5 (7.0, 80.0) *n* = 10	0.694	−2.0 (−12.5, 6.0) *n* = 25, *p* = 0.648	8.5 (−14.5, 79.0) *n* = 10, *p* = 0.275	−13.0 (−27.0, 1.0) *n* = 6, *p* = 0.178	−8.0 (−35.0, 5.0) *n* = 9, *p* = 0.236	**0.045**	0.112	0.267
**Functional Status**												
Borg’s Dyspnea Scale Score	7.0 (6.0, 8.0)	6.0 (5.5, 7.0)	6.5 (5.0, 8.0)	7.0 (6.0, 8.0)	0.478	−2.5 (−2.5, −2.0) *n* = 37, *p* < **0.001**	−2.0 (−3.0, −1.5) *n* = 11, *p* = **0.004**	−2.0 (−3.0, −1.5) *n* = 9, *p* = **0.013**	−2.5 (−3.0, −2.0) *n* = 16, *p* = **0.001**	0.678	0.589	0.456
Stair-Climbing Capacity	1.0 (0.5, 1.0) *n* = 37	1.0 (0.8, 1.5) *n* = 12	1.0 (1.0, 2.0) *n* = 10	1.0 (0.5, 1.0) *n* = 15	0.242	1.3 (1.0, 2.0) *n* = 35, *p* < **0.001**	2.0 (1.0, 3.0) *n* = 11, *p* = **0.009**	1.3 (1.0, 1.5) *n* = 9, *p* = **0.020**	1.0 (0.8, 2.0) *n* = 14, *p* = **0.002**	0.367	0.289	0.512

Note: Continuous variables are presented as median values (Q1, Q3). Changes are calculated as the difference between follow-up and baseline and presented as median values (95% CI) for continuous variables; *n* indicates non-missing paired observations (if different from group sample) *p*-values report the significance of changes within sample (group) over time. *p*-values for pairwise comparisons (benralizumab vs. dupilumab, benralizumab vs. mepolizumab, dupilumab vs. mepolizumab) are unadjusted and calculated using Dunn’s test with Holm’s correction applied. Results of post hoc analyses are reported using a letter-based system, where treatment groups sharing the same (or overlapped, e.g., A vs. AB, B vs. AB) uppercase letter do not differ significantly (*p* ≥ 0.05 after correction), and groups without overlapped (e.g., A vs. B) uppercase letters indicate significant differences (*p* < 0.05 after correction). Abbreviations: FEV1 = forced expiratory volume in 1 s; FVC = forced vital capacity; FeNO = fractional exhaled nitric oxide. Bold numbers indicate statistically significant values.

## Data Availability

All obtained and analyzed data are included in this article. Further enquiries can be directed to the corresponding author.

## References

[1] Hough K.P., Curtiss M.L., Blain T.J., Liu R.M., Trevor J., Deshane J.S., Thannickal V.J. (2020). Airway Remodeling in Asthma. Front. Med..

[2] Kuruvilla M.E., Lee F.E.H., Lee G.B. (2019). Understanding Asthma Phenotypes, Endotypes, and Mechanisms of Disease. Clin. Rev. Allergy Immunol..

[3] Dinarello C.A. (2011). Interleukin-1 in the Pathogenesis and Treatment of Inflammatory Diseases. Blood.

[4] Berry M.A., Hargadon B., Shelley M., Parker D., Shaw D.E., Green R.H., Bradding P., Brightling C.E., Wardlaw A.J., Pavord I.D. (2006). Evidence of a Role of Tumor Necrosis Factor α in Refractory Asthma. N. Engl. J. Med..

[5] Carr T.F., Kraft M. (2018). Use of Biomarkers to Identify Phenotypes and Endotypes of Severe Asthma. Ann. Allergy Asthma Immunol..

[6] Maspero J., Adir Y., Al-Ahmad M., Celis-Preciado C.A., Colodenco F.D., Giavina-Bianchi P., Lababidi H., Ledanois O., Mahoub B., Perng D.W. (2022). Type 2 Inflammation in Asthma and Other Airway Diseases. ERJ Open Res..

[7] McGregor M.C., Krings J.G., Nair P., Castro M. (2019). Role of Biologics in Asthma. Am. J. Respir. Crit. Care Med..

[8] Hirano T. (2021). IL-6 in Inflammation, Autoimmunity and Cancer. Int. Immunol..

[9] Peters M.C., McGrath K.W., Hawkins G.A., Hastie A.T., Levy B.D., Israel E., Phillips B.R., Mauger D.T., Comhair S.A., Erzurum S.C. (2016). Plasma Interleukin-6 Concentrations, Metabolic Dysfunction, and Asthma Severity: A Cross-Sectional Analysis of Two Cohorts. Lancet Respir. Med..

[10] Berry M., Brightling C., Pavord I., Wardlaw A. (2007). TNF-α in Asthma. Curr. Opin. Pharmacol..

[11] Brightling C., Berry M., Amrani Y. (2008). Targeting TNF-α: A Novel Therapeutic Approach for Asthma. J. Allergy Clin. Immunol..

[12] Lukacs N.W., Strieter R.M., Chensue S.W., Widmer M., Kunkel S.L. (1995). TNF-Alpha Mediates Recruitment of Neutrophils and Eosinophils during Airway Inflammation. J. Immunol..

[13] Elgueta R., Benson M.J., De Vries V.C., Wasiuk A., Guo Y., Noelle R.J. (2009). Molecular Mechanism and Function of CD40/CD40L Engagement in the Immune System. Immunol. Rev..

[14] Danese S., Fiocchi C. (2005). Platelet Activation and the CD40/CD40 Ligand Pathway: Mechanisms and Implications for Human Disease. Crit. Rev. Immunol..

[15] Kawabe T., Matsushima M., Hashimoto N., Imaizumi K., Hasegawa Y. (2011). CD40/CD40 ligand interactions in immune responses and pulmonary immunity. Nagoya J. Med. Sci..

[16] Saluk-Juszczak J., Królewska K. (2010). The Role of CD40/CD40L Pathway in the Biological Activity of Blood Platelets: Part I. Menopause Rev. Przegląd Menopauzalny.

[17] Hong G.U., Park B.S., Park J.W., Kim S.Y., Ro J.Y. (2013). IgE Production in CD40/CD40L Cross-Talk of B and Mast Cells and Mediator Release via TGase 2 in Mouse Allergic Asthma. Cell Signal.

[18] Li Y.J., Geng W.L., Li C.C., Wu J.H., Gao F., Wang Y. (2024). Progress of CCL20-CCR6 in the Airways: A Promising New Therapeutic Target. J. Inflamm..

[19] Chan D.I., Hunter H.N., Tack B.F., Vogel H.J. (2008). Human Macrophage Inflammatory Protein 3α: Protein and Peptide Nuclear Magnetic Resonance Solution Structures, Dimerization, Dynamics, and Anti-Infective Properties. Antimicrob. Agents Chemother..

[20] Shi Z.R., Mabuchi T., Riutta S.J., Wu X., Peterson F.C., Volkman B.F., Hwang S.T. (2023). The Chemokine, CCL20, and Its Receptor, CCR6, in the Pathogenesis and Treatment of Psoriasis and Psoriatic Arthritis. J. Psoriasis Psoriatic Arthritis.

[21] Dieu-Nosjean M.C., Massacrier C., Homey B., Vanbervliet B., Pin J.J., Vicari A., Lebecque S., Dezutter-Dambuyant C., Schmitt D., Zlotnik A. (2000). Macrophage Inflammatory Protein 3α Is Expressed at Inflamed Epithelial Surfaces and Is the Most Potent Chemokine Known in Attracting Langerhans Cell Precursors. J. Exp. Med..

[22] Reibman J., Hsu Y., Chen L.C., Bleck B., Gordon T. (2003). Airway Epithelial Cells Release MIP-3α/CCL20 in Response to Cytokines and Ambient Particulate Matter. Am. J. Respir. Cell Mol. Biol..

[23] Taylor A., Verhagen J., Blaser K., Akdis M., Akdis C.A. (2006). Mechanisms of Immune Suppression by Interleukin-10 and Transforming Growth Factor-β: The Role of T Regulatory Cells. Immunology.

[24] Tian T., Xie M., Sun G. (2024). Association of Systemic Immune-Inflammation Index with Asthma and Asthma-Related Events: A Cross-Sectional NHANES-Based Study. Front. Med..

[25] Tashiro H., Takahashi K., Kurihara Y., Sadamatsu H., Kimura S., Sueoka-Aragane N. (2021). Efficacy of Dupilumab and Biomarkers for Systemic Corticosteroid Naïve Allergic Bronchopulmonary Mycosis. Allergol. Int..

[26] Chen C.C., Buchheit K.M., Lee P.Y., Brodeur K.E., Sohail A., Cho L., Baloh C.H., Balestrieri B., Derakhshan T., Feng C. (2024). IL-4Rα Signaling Promotes Barrier-Altering Oncostatin M and IL-6 Production in Aspirin-Exacerbated Respiratory Disease. J. Allergy Clin. Immunol..

[27] Malik B., Bartlett N.W., Upham J.W., Nichol K.S., Harrington J., Wark P.A.B. (2023). Severe Asthma ILC2s Demonstrate Enhanced Proliferation That Is Modified by Biologics. Respirology.

[28] Rogliani P., Facciolo F., Melis E., Ritondo B.L., Gabriele M.C., Perduno A., Ora J., Calzetta L., Ora J. (2022). Pharmacological Characterization of the Anti-Inflammatory Effect of Mepolizumab and Benralizumab in a Human Ex Vivo Model of Asthma. Eur. Respir. J..

[29] Iyer S.S., Cheng G. (2012). Role of Interleukin 10 Transcriptional Regulation in Inflammation and Autoimmune Disease. Crit. Rev. Immunol..

[30] Ogawa Y., Duru E.A., Ameredes B.T. (2008). Role of IL-10 in the Resolution of Airway Inflammation. Curr. Mol. Med..

[31] Howell I., Yang F., Brown V., Cane J., Marchi E., Azim A., Busby J., McDowell P.J., Diver S.E., Borg C. (2024). Airway Proteomics Reveals Broad Residual Anti-Inflammatory Effects of Prednisolone in Mepolizumab-Treated Asthma. J. Allergy Clin. Immunol..

[32] Čelakovská J., Čermáková E., Boudková P., Krejsek J. (2025). Evaluation of the Levels of Interleukins IL-4, IL-13, IL-5, IL-10 and IL-33 in Atopic Dermatitis Patients with and without Dupilumab Therapy. Front. Immunol..

[33] Harb H., Chatila T.A. (2019). Mechanisms of Dupilumab. Clin. Exp. Allergy.

[34] Bergantini L., d’Alessandro M., Pianigiani T., Cekorja B., Bargagli E., Cameli P. (2023). Benralizumab Affects NK Cell Maturation and Proliferation in Severe Asthmatic Patients. Clin. Immunol..

[35] Lyakh L., Trinchieri G., Provezza L., Carra G., Gerosa F. (2008). Regulation of Interleukin-12/Interleukin-23 Production and the T-Helper 17 Response in Humans. Immunol. Rev..

[36] Gavett S.H., O’Hearn D.J., Li X., Huang S.K., Finkelman F.D., Wills-Karp M. (1995). Interleukin 12 Inhibits Antigen-Induced Airway Hyperresponsiveness, Inflammation, and Th2 Cytokine Expression in Mice. J. Exp. Med..

[37] Nonaka M., Ogihara N., Fukumoto A., Sakanushi A., Kusama K., Pawankar R., Yagi T. (2009). Synergistic Induction of Macrophage Inflammatory Protein-3α/CCL20 Production by Interleukin-17A and Tumor Necrosis Factor-α in Nasal Polyp Fibroblasts. World Allergy Organ. J..

[38] Newcomb D.C., Peebles R.S. (2013). Th17-Mediated Inflammation in Asthma. Curr. Opin. Immunol..

[39] Park S.Y., Kang M.J., Jin N., Lee S.Y., Lee Y.Y., Jo S., Eom J.Y., Han H., Chung S.I., Jang K. (2022). House Dust Mite-Induced Akt-ERK1/2-C/EBP Beta Pathway Triggers CCL20-Mediated Inflammation and Epithelial–Mesenchymal Transition for Airway Remodeling. FASEB J..

[40] Porsbjerg C., Rasmussen L., Nolte H., Backer V. (2007). Association of Airway Hyperresponsiveness with Reduced Quality of Life in Patients with Moderate to Severe Asthma. Ann. Allergy Asthma Immunol..

[41] Kim R.Y., Pinkerton J.W., Essilfie A.T., Robertson A.A.B., Baines K.J., Brown A.C., Mayall J.R., Ali M.K., Starkey M.R., Hansbro N.G. (2017). Role for NLRP3 Inflammasome-Mediated, IL-1β-Dependent Responses in Severe, Steroid-Resistant Asthma. Am. J. Respir. Crit. Care Med..

[42] Donnelly R.P., Young H.A., Rosenberg A.S. (2009). An Overview of Cytokines and Cytokine Antagonists as Therapeutic Agents. Ann. N. Y. Acad. Sci..

[43] Knight V., Sepiashvili L. (2025). Cytokine Testing and Challenges for Diagnostic and Clinical Monitoring Use. J. Allergy Clin. Immunol..

[44] Lou Y., Ke Q., Cui H., Shang Y., Yang C. (2021). Correlation Study of Cytokine Levels in Alveolar Lavage Fluid with Exhaled Nitric Oxide and Lung Function in Children with Bronchial Asthma. Transl. Pediatr..

[45] Ora J., De Marco P., Motta E., Laitano R., Calzetta L., Rogliani P. (2024). Real-World Efficacy of Biological Therapies in Severe Asthma: A Focus on Small Airways. J. Clin. Med..

[46] Niemiec-Górska A., Branicka O., Olszewska P., Mielcarska S., Glück J., Rymarczyk B., Gawlik R. (2025). The Comparative Effectiveness of Mepolizumab and Benralizumab in the Treatment of Eosinophilic Asthma. Adv. Respir. Med..

[47] Pelaia C., Giacalone A., Ippolito G., Pastore D., Maglio A., Piazzetta G.L., Lobello N., Lombardo N., Vatrella A., Pelaia G. (2024). Difficult-To-Treat and Severe Asthma: Can Real-World Studies on Effectiveness of Biological Treatments Change the Lives of Patients?. Pragmat. Obs. Res..

[48] Franceschi E., Drick N., Welte J.F.T., Suhling H., Santus P., Fischer B., Kayser M. (2023). The Impact of Anti-Eosinophilic Therapy on Exercise Capacity and Inspiratory Muscle Strength in Patients with Severe Asthma. ERJ Open Res..

[49] Panagiotou M., Koulouris N., Koutsoukou A., Rovina N. (2022). Daily Physical Activity in Asthma and the Effect of Mepolizumab Therapy. J. Pers. Med..

[50] Kai Y., Hishida Y. (2025). Dupilumab Treatment and 3-Dimensional Bronchial Tree Changes in Asthma-COPD Overlap. J. Allergy Clin. Immunol. Glob..

[51] Murugesan N., Saxena D., Dileep A., Adrish M., Hanania N.A. (2023). Update on the Role of FeNO in Asthma Management. Diagnostics.

[52] Watanabe H., Shirai T., Hirai K., Akamatsu T., Nakayasu H., Tamura K., Masuda T., Takahashi S., Tanaka Y., Kishimoto Y. (2022). Blood Eosinophil Count and FeNO to Predict Benralizumab Effectiveness in Real-Life Severe Asthma Patients. J. Asthma.

